# Relationship between threatened miscarriage and gestational diabetes mellitus

**DOI:** 10.1186/s12884-018-1955-2

**Published:** 2018-08-06

**Authors:** Hee Joong Lee, Errol Norwitz, Banghyun Lee

**Affiliations:** 1 Department of Obstetrics & Gynecology, College of Medicine, The Catholic University of Korea, Seoul, Republic of Korea; 2 Department of Obstetrics & Gynecology, Tufts University School of Medicine, Boston, MA USA; 3 Department of Obstetrics and Gynecology, Hallym University Kangdong Sacred Heart Hospital, 150, Seongan-ro, Gangdong-gu, Seoul, Republic of Korea

**Keywords:** Gestational diabetes mellitus, Threatened miscarriage, Borderline GDM, Maternal hyperglycemia

## Abstract

**Background:**

Both threatened miscarriage and gestational diabetes mellitus (GDM) are common complications of pregnancy. However, only one pilot study has reported that these complications are not related. We aimed to investigate whether threatened miscarriage is one of the risk factors of GDM.

**Methods:**

An unmatched case-control study of 1567 pregnant Korean women who underwent a two-step approach to diagnose GDM was retrospectively conducted. The eligible women were classified into normal (*n* = 840), borderline GDM (*n* = 480), and GDM (*n* = 247) groups. We analyzed the associations with threatened miscarriage in all groups with adjustment for confounding factors.

**Results:**

The proportion of women who experienced threatened miscarriage was significantly lower in the GDM group than in the normal group (adjusted odds ratio (OR), 0.38; 95% confidence interval (CI), 0.18–0.78). It was significantly lower in the maternal hyperglycemia group (borderline GDM and GDM groups) than in the normal group (adjusted OR, 0.66; 95% CI, 0.47–0.91). The proportion of women who experienced threatened miscarriage was also significantly lower in the GDM group than in the normal (adjusted OR, 0.35; 95% CI, 0.17–0.70) and borderline GDM groups (adjusted OR, 0.46; 95% CI, 0.22–0.94). Moreover, the proportion of women who experienced threatened miscarriage significantly decreased according to the severity of glucose intolerance (adjusted OR, 0.94; 95% CI, 0.76–1.16).

**Conclusion:**

This study demonstrates that threatened miscarriage is associated with decreased risk of GDM and the severity of glucose intolerance in Korean women. Additional studies are warranted to understand the pathophysiologic mechanisms that might exist between these frequent complications of pregnancy.

## Background

Threatened miscarriage, which is defined as any vaginal bleeding that occurs during the first half of a pregnancy, occurs in an estimated 14–20% of all pregnant women (14.8% in Korean women) [1, 2]. Approximately 50% of women who experienced threatened miscarriage eventually suffer miscarriages [3, 4]. Bleeding that originates from the uteroplacental vessels between the chorionic membrane and the uterine wall is often considered to be the most common cause of vaginal bleeding in threatened miscarriage [3]. Depending on the severity of bleeding, miscarriage can occur [3]. Many studies have reported that threatened miscarriage is associated with an increased risk of adverse obstetric outcomes, such as antepartum hemorrhage, preterm delivery, cesarean delivery, preterm premature rupture of membrane (PPROM), pregnancy-induced hypertension, preeclampsia, placenta previa, and placenta abruption, and adverse perinatal outcomes, such as perinatal death, small-for-gestational-age (SGA) infants and low birth weight [1, 3, 5, 6].

Maternal hyperglycemia occurs when insulin secretion from pancreatic β cells is inadequate for the increased insulin requirements during pregnancy [7]. Gestational diabetes mellitus (GDM) is defined as any severity of maternal hyperglycemia with onset or first recognition during pregnancy [8, 9]. However, a fasting plasma glucose level > 7.0 mmol/L or a casual plasma glucose > 11.1 mmol/L meets the threshold for type 2 diabetes diagnosis [8]. The prevalence of GDM ranges between 1 and 14% depending on the risk level of the affected population (5.7–9.5% in Korean women) [9–12]. Borderline GDM is usually considered to be maternal hyperglycemia that does not meet the diagnostic criteria for GDM and type 2 diabetes [10]. Although no clear consensus has been reached, 1-h venous plasma glucose ≥7.8 or 7.2 mmol/L on a positive 50-g oral glucose challenge test (OGCT) is considered a minimum glucose level for borderline GDM diagnosis [13, 14]. Several studies have reported that borderline GDM occurs in approximately 7–9% of all pregnant women [10].

Progesterone is essential for establishment and maintenance of pregnancy [15, 16]. Low progesterone levels are associated with an increased risk of first trimester miscarriage [17]. Recently, some studies reported that progesterone therapy during early pregnancy in women with threatened miscarriage may reduce pregnancy loss and the risk of adverse obstetric and perinatal outcomes [17–20]. Insulin resistance in pregnancy normally increases during the second and third trimesters [21]. Several studies in rats, mice, and adipocytes have reported that progesterone induces insulin resistance by multiple mechanisms during pregnancy [22–24]. The use of 17-alpha hydroxyprogesterone caproate for preterm delivery prevention is associated with an increased risk of developing GDM [25, 26]. Therefore, we hypothesized that women with threatened miscarriage possess lower levels of progesterone throughout pregnancy than those without threatened miscarriage. As a result, women with threatened miscarriage may have less insulin resistance during the second and third trimesters of pregnancy than those without threatened miscarriage, followed by a lower risk for glucose intolerance.

Both threatened miscarriage and GDM are common complications of pregnancy. Whether threatened miscarriage is associated with GDM warrants evaluation. To our knowledge, only one small-scale pilot study performed in Europe has reported that no relationship exists between threatened miscarriage and GDM [27]. Therefore, in the present study, we aimed to investigate whether threatened miscarriage is a risk factor of GDM and to evaluate this relationship according to the severity of glucose intolerance.

## Methods

This study was approved by the Institutional Review Board of The Catholic Medical Center at the Catholic University of Korea (No. XC14RIMI0013U) on February 1, 2014. Informed consent was waived. We reviewed the medical records of pregnant women who enrolled in the routine prenatal care program of Seoul or Uijeongbu St. Mary’s Hospital at the Catholic University of Korea before 12 weeks of gestation and who gave birth in the same hospital from January 1, 2006 to October 31, 2013. Pregnant women who underwent a two-step approach to diagnose GDM were included [7]. The exclusion criteria were as follows: women who did not undergo a 100-g oral glucose tolerance test (OGTT) after a positive 50-g OGCT; women who had other causes of vaginal bleeding (e.g., post-coital bleeding, polyp, vaginitis) at the time of assessment; women with fetal anomalies, multifetal gestation, or overt diabetes mellitus; and non-Korean ethnicity. Because only 3 non-Korean women met the study criteria, they were excluded from this study.

The medical records of 1567 pregnant women who were eligible for the study were reviewed. A total of 247 pregnant women were diagnosed with GDM. Borderline GDM and normal groups included 480 and 840 pregnant women, respectively.

In Seoul and Uijeongbu St. Mary’s Hospitals (the tertiary and secondary hospitals), the routine protocol to diagnose GDM was based on a two-step approach that consisted of a universal 50-g OGCT and a diagnostic 100-g OGTT [7]. A 50-g OGCT was performed at 24–28 weeks of gestation. Plasma glucose level > 7.8 mmol/L at 1-h post-glucose load without prior fasting was regarded as a positive GDM screening result, and a diagnostic 100-g OGTT was then recommended. In the diagnostic 100-g OGTT, the fasting plasma glucose levels were measured prior to glucose load ingestion, and three plasma glucose levels were measured at 1, 2, and 3 h following the glucose load ingestion. To diagnose GDM, we used the Carpenter and Coustan criteria, which included women with two or more plasma glucose measurements greater than the following thresholds: fasting glucose of ≥5.3 mmol/L, 1-h glucose of ≥10.0 mmol/L, 2-h glucose of ≥8.6 mmol/L, and 3-h glucose of ≥7.8 mmol/L [28]. Borderline GDM in women was defined as a positive OGCT and a negative diagnostic OGTT (in which plasma glucose measurements greater than the thresholds were absent or only one value was greater than the threshold). The pregnant women with GDM were managed with diet control, exercise, careful glucose monitoring, and medication (insulin therapy or oral hypoglycemic agents). In this study, 14.6% (*n* = 36) of the 247 women with GDM were managed with insulin (2 women were managed with oral hypoglycemic agents after insulin use for 2 months) and 85.4% (*n* = 211) were managed with diet control. The pregnant women with borderline GDM were managed with the same routine prenatal care as the healthy pregnant women, except for one woman who was administered with insulin.

Gestational age was calculated from the last menstrual period (LMP) and was confirmed by the crown-rump length (CRL), which was measured using ultrasound during the first trimester. If the CRL dating differed by more than 7 days from the LMP dating, gestational age was changed according to the CRL dating [27, 29]. Threatened miscarriage was defined as women whose medical records documented vaginal bleeding before 20 weeks of gestation [1]. Data regarding the amount of bleeding and the number of bleeding episodes were extracted from the medical records of the patients. Threatened miscarriage was classified as “light” if the bleeding was written as scanty or small amounts in the medical record; otherwise, it was classified as “heavy” [5]. Intrauterine hematoma was defined as a crescent-shaped echolucent area between the chorionic membranes and the placenta and/or the myometrium on ultrasound [1]. All eligible women were classified as either women who experienced threatened abortion (*n* = 194) or women who did not experience threatened miscarriage (*n* = 1373). One woman with GDM and two women with borderline GDM received weekly intramuscular injections of progesterone (250 mg, 1 to 3 times).

Pre-pregnancy body mass index (BMI) was calculated using the baseline weight (self-reported pre-pregnancy weight or weight measured at the first visit during early pregnancy when the pre-pregnancy weight was unknown) and measured height [14]. BMI was calculated as body mass in kg divided by height in m^2^. SGA (<10th percentile), appropriate for gestational age (AGA) (10–90th percentile), large for gestational age (LGA) (>90th percentile), and macrosomia (estimated fetal weight ≥ 4000 g) were defined for birth weight [30].

In this study, the association between threatened miscarriage and glucose intolerance was compared between the normal and GDM groups, between the normal and maternal hyperglycemia (borderline GDM and GDM groups) groups, and among the normal, borderline GDM, and GDM groups.

All analyses were performed using SAS software, version 9.2 (SAS Institute, Cary, NC, USA). To confirm that continuous variables were normally distributed, we applied the Shapiro-Wilk test. For non-normal distributed data, the significance of the differences between groups was tested using the Wilcoxon rank sum test. The categorical responses between the groups were analyzed using the chi-square test or Fisher’s exact test. The associations of the independent risk factors with each group were analyzed using logistic regression, with or without adjusting for confounding factors. Confounding factors included maternal age (< 35 years, ≥35 years), parity, history of prior GDM, gestational age at birth, and pre-pregnancy BMI, which are known risk factors for GDM [14, 31], and showed statistical significance in the current univariate analysis. According to a previous study, maternal age > 35 years was a risk factor for GDM [31]. Data were also adjusted for progesterone and insulin therapy based on their influence on the study result. Hosmer-Lemeshow goodness-of-fit test was performed (*P* > 0.05) to identify the most appropriate model. *P* < 0.05 was considered statistically significant.

## Results

The clinical characteristics of the study population are presented in Tables 1 and 2. The GDM group showed a lower incidence of intrauterine hematoma and a lower gestational age at birth than the normal group. The absolute maternal age and the rates of women with a maternal age ≥ 35 years, multiparity, preterm delivery, cesarean section, preeclampsia, history of prior GDM, pre-pregnancy BMI, and LGA were higher in the GDM group than in the normal group. The absolute birth weight and the incidence rates of operative delivery, premature rupture of membrane (PROM), PPROM, placenta previa, placenta abruption, stillbirth, SGA, and AGA, and macrosomia did not differ between the groups (Table 1). Additionally, the maternal hyperglycemia (borderline GDM and GDM groups) group exhibited a lower incidence of intrauterine hematoma and SGA, and a lower gestational age at birth than the normal group. The preterm delivery, cesarean section, preeclampsia, history of prior GDM, and pre-pregnancy BMI were higher in the maternal hyperglycemia (borderline GDM and GDM groups) group than in the normal group. Other variables were not different between the groups (Table 2).

**Table 1:** Clinical characteristics of the normal and GDM groups

	Total	Normal	GDM	*P* ^b^	OR (95% CI)	*P* ^c^
	(*n* = 1087)	(*n* = 840)	(*n* = 247)			
Maternal age (years), mean ± SD	34.1 ± 3.9	33.9 ± 3.9	34.7 ± 4.0	0.003	1.06 (1.02–1.10)	0.003
< 35, n (%)	623 (57.3)	498 (59.3)	125 (50.6)	0.015	ref	0.016
≥35, n (%)	464 (42.7)	342 (40.7)	122 (49.4)		1.42 (1.07–1.89)	
Parity, n (%)				0.003		0.003
Primipara	613 (56.4)	494 (58.8)	119 (48.2)		ref	
Multipara	474 (43.6)	346 (41.2)	128 (51.8)		1.54 (1.16–2.04)	
Intrauterine hematoma, n (%)	80 (7.4)	72 (8.6)	8 (3.2)	0.005	0.36 (0.17–0.75)	0.007
Gestational age at birth (weeks), mean ± SD	38.6 ± 1.6	38.8 ± 1.4	38.0 ± 2.0	< 0.001	0.74 (0.68–0.82)	< 0.001
Preterm delivery (before 37 weeks), n (%)	78 (7.2)	45 (5.4)	33 (13.4)	< 0.001	2.72 (1.70–4.38)	< 0.001
Delivery methods, n (%)				< 0.001		
Normal delivery	718 (66.1)	588 (70.0)	130 (52.6)		ref	
Operative delivery (vacuum)	12 (1.1)	10 (1.2)	2 (0.8)		0.91 (0.20–4.18)	0.90
Primary cesarean section	170 (15.6)	121 (14.4)	49 (19.8)		1.83 (1.25–2.69)	0.002
Repeat cesarean section	187 (17.2)	121 (14.4)	66 (26.7)		2.47 (1.73–3.52)	< 0.001
PROM, n (%)	186 (17.1)	148 (17.6)	38 (15.4)	0.41	0.85 (0.58–1.25)	0.41
PPROM, n (%)	38 (3.5)	25 (3.0)	13 (5.3)	0.09	1.81 (0.91–3.60)	0.09
Preeclampsia, n (%)	19 (1.8)	8 (1.0)	11 (4.5)	0.001	4.85 (1.93–12.19)	0.001
Placenta previa, n (%)	27 (2.5)	17 (2.0)	10 (4.0)	0.07	2.04 (0.92–4.52)	0.08
Placenta abruption, n (%)	7 (0.6)	7 (0.8)	0 (0.0)	0.36	0.35 (0.00–1.81)	0.33
Still birth, n (%)	3 (0.3)	3 (0.4)	0 (0.0)	> 0.999	0.88 (0.00–5.84)	0.92
History of prior GDM, n (%)	53 (4.9)	1 (0.1)	52 (21.1)	< 0.001	58.4 (37.73- > 999.99)	< 0.001
50 g OGCT (mmol/L), mean ± SD (*n* = 972) ^a^	124.4 ± 36.2	108.7 ± 17.1	172.1 ± 37.3	< 0.001	1.29 (1.23–1.36)	< 0.001
Pre-pregnancy BMI (kg/m^2^), mean ± SD	21.2 ± 3.3	20.5 ± 2.4	23.5 ± 4.4	< 0.001	1.34 (1.27–1.40)	< 0.001
Birth weight (g), mean ± SD	3188.2 ± 480.9	3189.3 ± 456.0	3184.4 ± 558.3	0.97	1.00 (0.99–1.00)	0.89
SGA, n (%)	140 (12.9)	114 (13.6)	26 (10.5)	0.21	0.75 (0.48–1.18)	0.21
AGA, n (%)	903 (83.1)	698 (83.1)	205 (83.0)	0.97	0.99 (0.68–1.45)	0.97
LGA, n (%)	44 (4.1)	28 (3.3)	16 (6.5)	0.028	2.01 (1.07–3.78)	0.030
Macrosomia, n (%)	43 (4.1)	29 (3.5)	14 (5.7)	0.12	1.68 (0.87–3.23)	0.12
Progesterone therapy	1 (0.1)	0 (0.0)	1 (0.4)	0.23	3.40 (0.18-Infinity)	0.46
Insulin therapy	36 (3.3)	0 (0.0)	36 (14.6)	< 0.001	203.42 (45.29-Infinity)	< 0.001

*SGA* small for gestational age: <10th percentile, *AGA* appropriate for gestational age: 10th–90th percentile, *LGA* large for gestational age: >90th percentile, *Macrosomia* Estimated fetal weight ≥ 4 kg [25]

^a^Missing data are excluded from statistical analyses

^b^Chi-square test or Fisher’s exact test for categorical variables and Wilcoxon rank sum test for continuous variables

^c^Univariate logistic regression

**Table 2:** Clinical characteristics of the normal and maternal hyperglycemia (Borderline GDM and GDM groups) groups

	Total	Normal	Borderline GDM and GDM	*P* ^b^	OR (95% CI)	*P* ^c^
	(*n* = 1567)	(*n* = 840)	(*n* = 727)			
Maternal age (years), mean ± SD	33.8 ± 3.9	33.9 ± 3.9	33.7 ± 4.0	0.23	0.99 (0.96–1.01)	0.35
< 35, n (%)	931 (59.4)	498 (59.3)	433 (59.6)	0.91	ref	0.91
≥35, n (%)	636 (40.6)	342 (40.7)	294 (40.4)		0.99 (0.81–1.21)	
Parity, n (%)				0.11		0.11
Primipara	892 (56.9)	494 (58.8)	398 (54.7)		ref	
Multipara	675 (43.1)	346 (41.2)	329 (45.3)		1.18 (0.97–1.44)	
Intrauterine hematoma, n (%)	113 (7.2)	72 (8.6)	41 (5.6)	0.025	0.64 (0.43–0.95)	0.026
Gestational age at birth (weeks), mean ± SD	38.5 ± 1.7	38.8 ± 1.4	38.2 ± 2.0	< 0.001	0.81 (0.76–0.86)	< 0.001
Preterm delivery (before 37 weeks), n (%)	123 (7.9)	45 (5.4)	78 (10.7)	< 0.001	2.12 (1.45–3.11)	< 0.001
Delivery methods, n (%)				< 0.001		
Normal delivery	1015 (64.8)	588 (70.0)	427 (58.7)		ref	
Operative delivery (vacuum)	22 (1.4)	10 (1.2)	12 (1.7)		1.65 (0.71–3.86)	0.25
Primary cesarean section	246 (15.7)	121 (14.4)	125 (17.2)		1.42 (1.08–1.88)	0.013
Repeat cesarean section	284 (18.1)	121 (14.4)	163 (22.4)		1.86 (1.42–2.42)	< 0.001
PROM, n (%)	267 (17.0)	148 (17.6)	119 (16.4)	0.51	0.92 (0.70–1.19)	0.51
PPROM, n (%)	54 (3.5)	25 (3.0)	29 (4.0)	0.27	1.35 (0.79–2.33)	0.28
Preeclampsia, n (%)	35 (2.2)	8 (1.0)	27 (3.7)	< 0.001	4.01 (1.81–8.89)	0.001
Placenta previa, n (%)	35 (2.2)	17 (2.0)	18 (2.5)	0.56	1.23 (0.63–2.41)	0.55
Placenta abruption, n (%)	10 (0.6)	7 (0.8)	3 (0.4)	0.36	0.49 (0.13–1.91)	0.31
Still birth, n (%)	4 (0.3)	3 (0.4)	1 (0.1)	0.63	0.39 (0.04–3.71)	0.41
History of prior GDM, n (%)	58 (3.7)	1 (0.1)	57 (7.8)	< 0.001	71.20 (9.86–514.24)	< 0.001
50 g OGCT (mmol/L), mean ± SD (*n* = 1445) ^a^	134.8 ± 34.3	108.7 ± 17.1	161.5 ± 25.9	< 0.001	1.42 (1.34–1.50)	< 0.001
Pre-pregnancy BMI (kg/m^2^), mean ± SD	21.2 ± 3.3	20.5 ± 2.4	22.1 ± 4.0	< 0.001	1.18 (1.14–1.23)	< 0.001
Birth weight (g), mean ± SD	3186.7 ± 485.0	3189.3 ± 456.0	3183.6 ± 516.7	0.82	1.00 (0.99–1.00)	0.82
SGA, n (%)	187 (11.9)	114 (13.6)	73 (10.0)	0.032	0.71 (0.52–0.97)	0.032
AGA, n (%)	1319 (84.2)	698 (83.1)	621 (85.4)	0.21	1.19 (0.91–1.57)	0.21
LGA, n (%)	61 (3.9)	28 (3.3)	33 (4.5)	0.22	1.38 (0.83–2.31)	0.22
Macrosomia, n (%)	59 (3.8)	29 (3.5)	30 (4.1)	0.48	1.20 (0.72–2.03)	0.48
Progesterone therapy	3 (0.2)	0 (0.0)	3 (0.4)	0.10	4.46 (0.68-Infinity)	0.20
Insulin therapy	37 (2.4)	0 (0.0)	37 (5.1)	< 0.001	64.23 (14.36-Infinity)	< 0.001

*SGA* small for gestational age: <10th percentile, *AGA* appropriate for gestational age: 10th–90th percentile, *LGA* large for gestational age: >90th percentile, *Macrosomia* Estimated fetal weight ≥ 4 kg [25]

^a^Missing data are excluded from statistical analyses

^b^Chi-square test or Fisher’s exact test for categorical variables and Wilcoxon rank sum test for continuous variables

^c^Univariate logistic regression

The proportion of women who experienced threatened miscarriage was significantly lower in the GDM group than in the normal group with or without adjusting for confounding factors (adjusted odds ratio (OR), 0.38; 95% confidence interval (CI), 0.18–0.78; *P* = 0.009) (Table 3). It was also significantly lower in the maternal hyperglycemia (borderline GDM and GDM groups) group than in the normal group (adjusted OR, 0.66; 95% CI, 0.47–0.91; *P* = 0.013) (Table 4). The amount of bleeding and the number of bleeding episodes in women who experienced threatened miscarriage were not different between the groups (Tables 3 and 4).

**Table 3:** Association between GDM and threatened miscarriage

	Total (*n* = 1087)	Normal (*n* = 840)	GDM (*n* = 247)	OR (95% CI)	*p*	Adjusted OR (95% CI)	*p*
Threatened miscarriage, n (%)					0.002		0.009
Negative	949 (87.3)	719 (85.6)	230 (93.1)	ref		ref	
Positive	138 (12.7)	121 (14.4)	17 (6.9)	0.44 (0.26–0.75)		0.38 (0.18–0.78)	
Amount of bleeding, n (%) (*n* = 137) ^a^					0.11		0.69
Light	117 (85.4)	100 (83.3)	17 (100.0)	ref		ref	
Heavy	20 (14.6)	20 (16.7)	0 (0.0)	0.21 (0.00–1.05)		0.63 (0.00–3.46)	
Number of bleeding episodes, n (%) (*n* = 137) ^a^					0.13		0.53
≤ 2	108 (78.8)	92 (76.7)	16 (94.1)	ref		ref	
≥ 3	29 (21.2)	28 (23.3)	1 (5.9)	0.21 (0.03–1.62)		0.50 (0.06–4.42)	

Statistical analyses were performed by univariable and multivariable logistic regression

The data were adjusted for maternal age (< 35 years, ≥35 years), parity, history of prior GDM, pre-pregnancy BMI, progesterone therapy, and insulin therapy

^a^Missing data are excluded from statistical analyses

**Table 4:** Association between maternal hyperglycemia (Borderline GDM and GDM) and threatened miscarriage

	Total (*n* = 1567)	Normal (*n* = 840)	Borderline GDM and GDM (*n* = 727)	OR (95% CI)	*p*	Adjusted OR (95% CI)	*p*
Threatened miscarriage, n (%)					0.009		0.013
Negative	1373 (87.6)	719 (85.6)	654 (90.0)	ref		ref	
Positive	194 (12.4)	121 (14.4)	73 (10.0)	0.66 (0.49–0.90)		0.66 (0.47–0.91)	
Amount of bleeding, n (%) (*n* = 192) ^a^					0.29		0.15
Light	164 (85.4)	100 (83.3)	64 (88.9)	ref		ref	
Heavy	28 (14.6)	20 (16.7)	8 (11.1)	0.63 (0.26–1.50)		0.66 (0.26–1.67)	
Number of bleeding episodes, n (%) (*n* = 192) ^a^					0.022		0.14
≤ 2	157 (81.8)	92 (76.7)	65 (90.3)	ref		ref	
≥ 3	35 (18.2)	28 (23.3)	7 (9.7)	0.35 (0.15–0.86)		0.36 (0.14–0.93)	

Statistical analyses were performed by univariable and multivariable logistic regression

The data were adjusted for maternal age (< 35 years, ≥35 years), parity, history of prior GDM, pre-pregnancy BMI, progesterone therapy, and insulin therapy

^a^Missing data are excluded from statistical analyses

Threatened miscarriage showed significant associations among the normal, borderline GDM, and GDM groups. The proportion of women who experienced threatened miscarriage was significantly lower in the GDM group than in the normal group with or without adjusting for confounding factors (adjusted OR, 0.35; 95% CI, 0.17–0.70; *P* = 0.003). The proportion of women who experienced threatened miscarriage was also significantly lower in the GDM group than in the borderline GDM group with or without adjusting for confounding factors (adjusted OR, 0.46; 95% CI, 0.22–0.94; *P* = 0.034). Moreover, the proportion of women who experienced threatened miscarriage significantly decreased according to the severity of glucose intolerance with or without adjusting for confounding factors (adjusted OR, 0.94; 95% CI, 0.76–1.16; *P* < 0.001) (Tables 5 and 6).

**Table 5:** Association between the severity of maternal hyperglycemia and threatened miscarriage: Multinomial logistic regression

	Normal (*n* = 840)	Borderline GDM (*n* = 480)	GDM (*n* = 247)	OR (95% CI)				Adjusted OR (95% CI)			
				Normal vs GDM	*p*	Borderline GDM vs GDM	*p*	Normal vs GDM	*p*	Borderline GDM vs GDM	*p*
Threatened miscarriage					0.002		0.044		0.003		0.034
Negative	719 (85.6)	424 (88.3)	230 (93.1)	ref		ref		ref		ref	
Positive	121 (14.4)	56 (11.7)	17 (6.9)	0.44 (0.26–0.75)		0.56 (0.32–0.99)		0.35 (0.17–0.70)		0.46 (0.22–0.94)	

Statistical analyses were performed by univariable and multivariable logistic regression

The data were adjusted for maternal age (< 35 years, ≥35 years), parity, history of prior GDM, pre-pregnancy BMI, progesterone therapy, and insulin therapy

**Table 6:** Association between the severity of maternal hyperglycemia and threatened miscarriage: Ordinal logistic regression

	Normal (*n* = 840)	Borderline GDM (*n* = 480)	GDM (*n* = 247)	OR (95% CI)	*p*	Adjusted OR (95% CI)	*p*
Threatened miscarriage					0.003		< 0.001
Negative	719 (85.6)	424 (88.3)	230 (93.1)	ref		ref	
Positive	121 (14.4)	56 (11.7)	17 (6.9)	0.63 (0.47–0.86)		0.94 (0.76–1.16)	

Statistical analyses were performed by univariable and multivariable logistic regression

The data were adjusted for maternal age (< 35 years, ≥35 years), parity, history of prior GDM, pre-pregnancy BMI, progesterone therapy, and insulin therapy

## Discussion

In this case-control study, we found that the women who experienced threatened miscarriage had a significantly decreased risk of GDM in current pregnancies. This finding was observed in the comparison between the normal and GDM groups, between the normal and maternal hyperglycemia (borderline GDM and GDM groups) groups, and among the normal, borderline GDM, and GDM groups. Moreover, threatened miscarriage showed an inverse correlation with the severity of glucose intolerance. Our data supported our initial hypothesis that low progesterone levels in women with threatened miscarriage might be associated with a low increase in insulin resistance during the second and third trimesters of pregnancy, followed by a low incidence of glucose intolerance. Our findings potentially aids in understanding these common complications of pregnancy. However, future prospective, large-scale studies are necessary to explore the relationship between threatened miscarriage and GDM.

A prospective pilot study based on a small population with a very low incidence of GDM [threatened miscarriage vs normal pregnancy, 3/69 (4.3%) vs 11/564 (2.0%), respectively] reported no relationship between threatened miscarriage and GDM [27]. By contrast, our study (normal pregnancy vs GDM with or without borderline GDM) retrospectively showed relatively lower incidences of GDM and maternal hyperglycemia (borderline GDM and GDM) in women who experienced threatened miscarriage [27]. However, in a large-scale retrospective study involving women who received intramuscular high-dosage progesterone therapy (the total accumulated dose was ≥500 mg) due to threatened miscarriage in the second and third months of pregnancy, the incidence of GDM was slightly higher in women with threatened miscarriage [37/532 (6.95%)] than in women with normal pregnancy [1141/21,054 (5.42%)], but it was not statistically significant [20]. In the same study, threatened miscarriage was not associated with an increased risk of adverse obstetric and perinatal outcomes, such as preterm delivery, hypertensive disorders in pregnancy, placenta previa, placenta abruption, live births, and low birth weight, compared with normal pregnancy [20]. These findings demonstrate that the risks of common adverse obstetric and perinatal outcomes of threatened miscarriage were affected by progesterone therapy. In particular, considering the effect of progesterone therapy, we speculate that threatened miscarriage in the previous study might be associated with a low risk of GDM, which corresponded with the results of our study that showed inverse correlation between threatened miscarriage and GDM (Tables 3, 5 and 6).

Intrauterine hematoma is detected in 18–39% of pregnant women who experience threatened miscarriage. It is associated with an increased risk of adverse obstetric outcomes, such as preterm delivery, pregnancy-induced hypertension, preeclampsia, placental abruption, and SGA, through mechanisms that are similar to those that result in threatened miscarriage [1, 3]. In the present study, the incidence of intrauterine hematoma (50.5%) in women with threatened miscarriage was higher than that described in previous reports [3]. Moreover, the incidence of intrauterine hematoma decreased in women with GDM and maternal hyperglycemia (borderline GDM and GDM) compared with the normal women, in accordance with the risk of threatened miscarriage (Tables 1 and 2).

The amount of vaginal bleeding during pregnancy is a subjective measurement. Consequently, distinguishing between light and heavy vaginal bleeding has not been effective [6]. In our study, although the data did not reach statistical significance, GDM and maternal hyperglycemia (borderline GDM and GDM) occurred less frequently in women with heavy bleeding than in women with light bleeding. They also occurred less frequently in women with higher bleeding levels (≥3) than in women with lower bleeding levels (≤2), which is in accordance with the risk of threatened miscarriage (Tables 3 and 4).

In our study, the GDM group displayed typical characteristics of women with GDM compared with the normal group, which is in accordance with prior reports (Table 1) [30–33]. However, compared with previous reports [34–36], we detected a similar birth weight, low pre-pregnancy BMI and rates of macrosomia/LGA, and a high rate of SGA in the study cohort. This result suggests inherent ethnic differences in which Koreans consider lean body shape as superior to obese, followed by excessive diet control and exercise (Table 1). Additionally, in contrast to prior studies that reported higher birth weight, higher macrosomia/LGA rates, and lower SGA rates in women with GDM [35–37], the birth weight and the incidence rate of SGA, LGA, or macrosomia were unchanged in women with GDM and maternal hyperglycemia (borderline GDM and GDM) compared with the normal women in our study (Tables 1 and 2). These findings could be explained by the low pre-pregnancy BMI and the low rates of macrosomia/LGA in our study groups in addition to the higher preterm delivery rate and the shorter gestational age at birth in women with GDM and maternal hyperglycemia (borderline GDM and GDM) compared with the normal women.

The significance of our study might be limited because definite mechanisms to elucidate the relationship between threatened miscarriage, and GDM and the severity of glucose intolerance were not clinically investigated. However, our study is important because it focused on their specific relationship, although it was performed using a retrospective design.

## Conclusions

In this retrospective study, we demonstrated that GDM was less frequently observed in Korean women who experienced threatened miscarriage showing inverse relationship between threatened miscarriage and the severity of glucose intolerance. Further research is recommended to confirm these relationships and to evaluate the pathophysiologic mechanisms that interplay between these common obstetric complications. We believe that these interesting findings will help improved care for women with adverse obstetric outcomes.

## Abbreviations

AGA: Appropriate for gestational age
BMI: Body mass index
CRL: Crown-rump length
GDM: Gestational diabetes mellitus
LGA: Large for gestational age
LMP: Last menstrual period
OGCT: Oral glucose challenge test
OGTT: Oral glucose tolerance test
PPROM: Preterm premature rupture of membrane
SGA: Small for gestational age
